# Role of Heat Shock Proteins (HSP70 and HSP90) in Viral Infection

**DOI:** 10.3390/ijms22179366

**Published:** 2021-08-29

**Authors:** Anna Lubkowska, Waldemar Pluta, Aleksandra Strońska, Alicja Lalko

**Affiliations:** 1 Department of Functional Diagnostics and Physical Medicine, Pomeranian Medical University in Szczecin, Żołnierska 54, 71-210 Szczecin, Poland; waldemar.pluta@pum.edu.pl; 2 Department of Pharmacognosy and Natural Medicines, Pomeranian Medical University in Szczecin, Powstańców Wlkp. 72, 70-111 Szczecin, Poland; aleksandra.stronska@pum.edu.pl; 3 Student Research at the Chair and Department of Functional Diagnostics and Physical Medicine, Pomeranian Medical University, Żołnierska 54, 71-210 Szczecin, Poland; alicjalalko@gmail.com

**Keywords:** HSP70, HSP90, viral infection

## Abstract

Heat shock proteins (HSPs) are a large group of chaperones found in most eukaryotes and bacteria. They are responsible for the correct protein folding, protection of the cell against stressors, presenting immune and inflammatory cytokines; furthermore, they are important factors in regulating cell differentiation, survival and death. Although the biological function of HSPs is to maintain cell homeostasis, some of them can be used by viruses both to fold their proteins and increase the chances of survival in unfavorable host conditions. Folding viral proteins as well as replicating many different viruses are carried out by, among others, proteins from the HSP70 and HSP90 families. In some cases, the HSP70 family proteins directly interact with viral polymerase to enhance viral replication or they can facilitate the formation of a viral replication complex and/or maintain the stability of complex proteins. It is known that HSP90 is important for the expression of viral genes at both the transcriptional and the translational levels. Both of these HSPs can form a complex with HSP90 and, consequently, facilitate the entry of the virus into the cell. Current studies have shown the biological significance of HSPs in the course of infection SARS-CoV-2. A comprehensive understanding of chaperone use during viral infection will provide new insight into viral replication mechanisms and therapeutic potential. The aim of this study is to describe the molecular basis of HSP70 and HSP90 participation in some viral infections and the potential use of these proteins in antiviral therapy.

## 1. Introduction

The discovery of Heat Shock Proteins (HSPs) is attributed to the Italian geneticist Ferruccio Ritossa [1], who during his research mistakenly increased the incubation temperature of *Drosophila melanogaster* larvae, which resulted in an increased transcription of genes of proteins unknown at that time. Less than 10 years later it was noticed that the proteins discovered by Ritossa prevented damage to cells which were associated with abnormal proteins resulting from heat shock [2]. Later studies have shown that HSP can be induced not only by temperature but also by other stressors, including starvation, hypoxia, physiological or environmental attacks, chemical or UV exposure and the invasion of pathogens [3].

The division of HSP proteins is based on their molecular weight: there are small HSPs (sHSP), i.e., HSP21 and HSP10, medium HSPs, i.e., HSP60 and HSP70, and large HSPs, i.e., HSP90 and HSP100. Chaperones of larger molecular sizes than 60 kDa are ATP-dependent and utilize their enzymatic activity to induce conformational changes in the client polypeptide by hydrolyzing adenosine triphosphate (ATP) to adenosine diphosphate (ADP). In terms of expression, two types of HSP are distinguished: stress-induced isomers, the expression level of which depends on the presence of a stressor and native forms with constant, stress-independent expression [4,5]. HSPs are located in various cell structures, including the nucleus, mitochondria, chloroplasts, endoplasmic reticulum and cytosol in all types of prokaryotes and eukaryotes [3]. They are expressed during the cell cycle, embryogenesis and differentiation, and are stimulated by growth factors [6].

Their main role is to protect cells against stressors by participating in the correct folding of newly formed proteins, repairing misfolded proteins and by participating in the transport of proteins to their site of action [7]. HSPs usually make up about 5–10% of the total protein in most cells but their intracellular concentration may increase within a few minutes under the influence of stressors up to 20 times in order to suppress or attenuate undesired effects [8,9].

In physiological conditions and in the absence of stressors, HSPs are associated with heat shock factors (HSF), especially HSF-1. An increase in the number of damaged proteins causes the detachment of HSP from HSF in order to bind the damaged protein. Consequently, there is an increase in the number of free HSFs in the cell, which function as transcription factors and bind to heat shock DNA elements, triggering the expression of HSP, a so-called heat shock response HSR. This increase in HSP expression means that free HSF is rebound, reducing HSP gene transcription and inhibiting HSF binding to DNA. This approach enables HSPs to independently regulate their own expression. During cellular stress, the level of HSP in the cytoplasm increases and their transport takes place mainly to the cell nucleus, where they protect DNA, pre-mRNA, pre-ribosomal and nuclear proteins against damage and degradation. They also participate in the activation of specific genes [7].

Although HSPs are protective proteins, they can be turned against the organism in which they are found. Viruses lacking their HSPs use the host’s HSP to fold their proteins and increase the effectiveness of infection. A better understanding of the mechanisms of viral infections in which HSPs participate would allow for the development of more effective antiviral drugs based on these proteins. HSP70 and HSP90 inhibitors show great therapeutic potential. The aim of this review is to present the latest reports on the role of HSP70 and HSP90 in viral infections and to present these proteins as an effective therapeutic target.

## 2. Characteristics of Selected HSP’s

### 2.1. HSP70

As one of the most ubiquitous molecular chaperones located in all the cellular compartments of eukaryotes, HSP70s regulate every aspect of cellular proteostasis, including: the folding of both nascent and misfolded proteins; protein assembly and the regulation of their activity; the translocation into mitochondria, chloroplasts and the endoplasmic reticulum; the degradation of and prevention from dismantling protein aggregates [10,11]. HSP70 is composed of two large, functional domains: the N-terminal nucleotide-binding domain (NBD) and the C-terminal substrate-binding domain (SBD). The NBD is about 44 kDa and provides energy to power HSP70 activity by binding and hydrolyzing ATP to ADP. It is divided into two lobes (I and II), which are further divided into two subdomain regions (IA, IB and IIA, IIB). The characteristic structure of subdomains forms a V-shaped cleft where the nucleotide binds. In turn, SBD (25 kDa) is a tweezers-like binding site for polypeptide substrates and is divided into two domains called α and β. A 12-kDa β-SBD is organized such as a sandwich and is made up of two antiparallel β-sheets of four strands each. The β-domain contains the substrate-binding site used for binding misfolded proteins which is located between the two β sheets. The substrate-binding pocket is highly hydrophobic and displays a high affinity for hydrophobic substituents such as Leu. α-SBD is a 15-kDa α-helical subdomain which forms a lid that covers the substrate-binding cleft and stabilizes the loop regions of the β-subdomain. The domains are connected by the interdomain linker, which is composed of 10–12 highly conserved hydrophobic amino acids which assume an unstructured conformation in the ADP-bound state and a β-stranded conformation in the ATP-bound state [10,12,13]. SBD is similar in shape to tweezers, allowing HSP70 to interact with polypeptides and protein complexes regardless of their size. Moreover, this domain can stabilize these units by interacting with folding intermediators and folded pieces of proteins. Interaction with folded proteins is not constant and is regulated by an allosteric mechanism. It involves the hydrolysis of ATP and the rebinding of ATP in NBD with the binding and release of substrate polypeptides. The allosteric mechanism of HSP70 can be modified by J domain proteins (JDP) and nucleotide exchange factors (NEF) [14].

Such a wide spectrum of activity of HSP70s proves that these proteins, in order to adapt to the conditions of growth and stress in the cell, are able to act on a variety of substrates [11]. There are at least 14 proteins within the human HSP70 family, which differ in terms of expression level, amino acid composition and subcellular localization. Proteins can be stress induced (e.g., HSPA1 and HSP6) or constitutively expressed (e.g., HSPA5, HSPA8 and HSPA9). The chaperone activity of a protein consists of the hydrolysis of ATP. The interaction with unfolded protein begins with the binding of HSP70 to its substrates. The initially low affinity increases significantly after ATP is hydrolyzed to ADP by the cochaperone HSP40. Finally, with the help of nucleotide exchange factors, ADP dissociates to reset the cycle [15]. The most important functions of the HSP70 isoforms are presented in Table 1.

### 2.2. HSP90

A characteristic feature of all HSP90 family proteins is the identical domain structure. It consists of the N-terminal domain (NTD), the middle domain (M domain) and the carboxy-terminal domain (CTD). NTD contains in its structure an ATP binding site that is essential for the ATPase activity of HSP90. It is required for the chaperone cycle and protein binding of the HSP90 client [32,33]. The MD domain has a characteristic way of controlling the functions of HSP90 through the binding of phosphate to the ATP specified for NTD. Thus, the MD domain influences the ATPase activity of HSP90 [34]. There are two key sites in the CTD domain: one for calmodulin binding [35], and the other for HSP9 homodimerization [36]. In addition, there is a nucleotide binding site that opens after taking the N-terminal site and serves as an allosteric regulator of N-terminal ATPase activity [37].

During the ATPase cycle, HSP90 undergoes various transformations of conformational states. The first intermediate state is the binding of ATP to NTD, which leads to the closure of the ATP cover over the bound nucleotide. The subsequent conformation changes lead to the dimerized state at the amino terminus (closed state 1) with replaced segments in NTD [38] and compacting the distance between MD and NTD (closed state 2), as a result of which ATPase has a competent conformation. The HSP90 chaperone cycle ends with the release of ADP and phosphate and the dissociation of the amino terminus [39,40,41].

Heat shock factor 1 (HSF1) is mainly responsible for the expression of HSP90 at the transcription stage. Its functioning is based on the regulation of the heat shock response (HSR) in eukaryotic cells [42,43]. HSF1 is also the major regulator of HSP90 levels in cells. This large influence of HSF1 on HSP90 resulted in the development of a complex series of regulatory mechanisms, including transcription, HSF1 trimerizations, cooperative binding to HSE, post-translational modifications and the ability of HSF1 to directly detect stress. All these mechanisms integrate different signals, resulting in a change in HSP90 concentration in cells [44].

The functions of HSP90 are modulated by numerous post-translational modifications. These include acetylation, phosphorylation, sumoylation and S-nitrosylation. Certain changes in the acetylation state are key regulators of co-chaperone binding [45], including HSP90 hyperacetylation, causing the inability to bind to co-chaperone p23, loss of guardian activity and impaired activation of the glucocorticoid receptor (GR) [46,47]. Phosphorylation has been shown to slow down the conformational cycle of HSP90 and to influence client maturation and interactions with the co-chaperone [48]. Protein phosphatase PP5 is the co-chaperone of HSP90 that affects the phosphorylation status. In the absence of PP5, a state of hyperphosphorylation occurs, which negatively affects the maturation of the client [49]. S-nitrosylation of HSP90 in CTD inhibits the ATPase reaction and reduces the activating effect of HSP90 on client endothelial nitric oxide synthase (eNOS). It can be concluded that there is an eNOS feedback mechanism [50]. S-nitrosylation affects the ATPase activity of HSP90 as well as its chaperone activity [51]. The most important functions of the HSP90 isoforms are presented in Table 2.

The determinants of HSP90 customers are still the subject of many researches and discussions. It is still not entirely clear how HSP90 recruits its customers, but it is known that they are not aware of any common binding patterns (e.g., hydrophobic sequences) [52].

## 3. The Role of HSP70 and HSP90 in Viral Infections

### 3.1. RNA Viruses

Table 3 shows stages of infection of RNA viruses, which use the HSP70 and/or HSP90 host. The following subsection focuses on viruses in which both HSP70 and HSP90 are involved.

#### 3.1.1. Human Enterovirus (EV-71)

Human enterovirus 71 (EV-71) is a virus of the Enterovirus genus in the *Picornaviridae* family. EV-71 has a positive sense single-stranded RNA genome. It is approximately 7400 bp in length. Most often it causes large outbreaks of hand-foot-and-mouth disease (HFMD), as well as aseptic meningitis and encephalitis. EV-71 is stated to be responsible for the epidemics of severe neurological diseases in Asia, Europe and the USA [104,105].

In the research of Xu et al. [79] it is shown that HSP70 present on the surface of host cells can act as a site of attachment for EV-71. This allows for the initial binding of EV-71 to the host cells and subsequent infection, though the amount of viral cells attached to the host cell surface may not only determine the effectiveness of the infection. These studies also provided information that HSP70 established an additional route for EV-71 entry into host cells. The chaperone knockdown with lipofectamine 2000 did not eliminate the infection. This may indicate that the interaction of the chaperone protein with EV-71 enables the promotion of the attachment of the virus to other viral binding cell receptors, and then enhances viral endocytosis in the host cells.

More detailed studies on the contribution of proteins from the HSP70 family were carried out by Yu-Siang et al. [106]. The study analyzed the role of HSPA1, HSPA8 and HSPA9 at each stage of the EV-71 replication cycle. The results proved that the studied proteins are involved in all phases of the virus life cycle. Deficiency in any of the proteins in the HSP70 subnetwork leads to a significant reduction in viral internalization. The entry of EV-71 into the cells takes place through endocytosis via the scavenger receptor class B member 2 (SCARB2), which depends on clathrin and dynamin 2. HSPA8 has the ability to separate the clathrin triskelion from vesicles covered with clathrin; thus, influencing the internation of the virus. The current knowledge regarding the effects of HSPA1 and HSPA9 in promoting the entry of EV-71 into host cells is limited.

Yueh-Liang Tsou et al. [80] showed the direct interaction of HSP90 with EV-71 on the cell surface. The use of HSP90 inhibitors such as geldanamycin (GA), siRNA or antibodies, disrupted the entry of the virus at an early stage. In addition, viral replication was disrupted and the stability of the capsid proteins was impaired at the post-translational level. Following treatment with inhibitors, the newly synthesized capsid proteins lost the ability to assemble properly in the infected cell.

#### 3.1.2. Dengue Virus (DENV)

Dengue virus (DENV) is a virus from the *Flaviviridae* family of the *Flavivirus* genus. DENV is an enveloped, single-stranded, positive RNA virus of approximately 11 kb [107]. The virus is transmitted by female *Aedes aegypti* mosquitoes and causes hemorrhagic fever. The spectrum of clinical disease can vary from asymptomatic to a wide range of syndromes with severe clinical symptoms [108].

Jorge Reyes-del Valle et al. [76] suggested that HSP90 and HSP70 are involved in DENV entry into host cells. They proved this by using antibodies against HSP90 and HSP70 in an infection inhibition assay. The results show that anti-HSP90 and anti-HSP70 antibodies block DENV infectivity in neuroblastoma cells. Additionally, both HSP90 and HSP70 associate with membrane lipid rafts in response to DENV infection.

Several studies have shown that HSP70 acts as permeable DENV receptor in mammalian and mosquito cells. HSPA5 (or otherwise called GRP78), a member of the HSP70 family, was identified as the DENV receptor in the HepG2 cell line by a viral overlay protein (VOPBA) binding assay coupled with mass spectrometry. The use of a specific antibody directed against the N-terminal domain of GRP78 leads to the blockage of this protein on the cell surface, resulting in a reduction in DENV production. On the other hand, the use of an inhibitor that binds to the C-terminal domain results in a higher viral production depending on the dose used [109].

Heat shock stress has been shown to enhance replication in the U937 cell line by increasing the expression of HSP70 and HSP90 on the cell surface, especially in lipid rafter microdomains. Interestingly, the increase in HSP70 and HSP90 expression induced by heat stress did not result in increased DENV binding, but facilitated viral entry. The findings suggest that HSP70 and HSP90 are necessary for DENV entry into the cell, and that heat shock stress is optimal for cell replication in the U937 cell line [110].

Research by Taguwa et al. reports that HSP70 is essential for the life cycle of DENV, including virus entry, virus replication and virion production. In order to confirm the role of HSP70 in virus entry into the cell, they used a specific HSP70 inhibitor—JG40. Treatment of cells with this inhibitor reduced viral RNA [111].

Studies by Howe et al. [112] report that the amount of inducible HSP70 increases during DENV infection and is used by the virus during its entry into the host cell. Interestingly, the use of an HS-10 inhibitor specific for HSP90 also increases the level of inducible HSP70 on the surface of infected cells, which in turn results in increased infectivity in the U937 + DC-SIGN cell line. It is shown that the use of an HS-72 inhibitor specific for inducible HSP70 before infection reduces the infectivity of DENV [113].

#### 3.1.3. Influenza A Virus (IAV)

The influenza A virus (IAV) genome consists of eight segmented, negative RNAs. Viral RNA (vRNA) together with viral RNA-dependent RNA polymerase subunits—PB1, PB2 and PA—and NP nucleoprotein form viral ribonucleoprotein (vRNP) complexes [114].

HSP90 stabilizes viral polymerase activity by binding to its PB2 subunit. HSP90 is transported to the cell nucleus with PB2 or the PB1–PB2 complex. The HSP90–PB2 complex interacts with PB1–PA in the cell nucleus where a ternary complex is formed and HSP90 is released from PB2. The accumulation of HSP90 in the nucleus through interaction with viral polymerases is needed for the regulation of RNA virus synthesis in the nucleus [88,89].

In addition to the role of HSP90 in the stabilization of viral polymerase and in nuclear transport, HSP90 promotes influenza A-mediated apoptosis. It activates the caspase cascade, essential for viral replication, pathogenesis and virulence [38,39,40,41]. HSP90 has also been shown to counteract the degradation of influenza A (NA) neuraminidase and increase its stability [42,43]. The formation of the HSP90-NA complex increases the viability of the cells, which leads to more virus production [115].

Studies have shown that HSP70 can inhibit IAV replication by disrupting viral polymerase binding to viral RNA [116] or by preventing the RNP complex from being exported to the nucleus [103].

In addition, studies by Manzoor et al. proved that HSP70 interacts and translocates into the nucleus with PB2 monomers or PB2/PB1 heterodimers, which, consequently, may aid viral polymerase complex formation. They also confirmed that the precipitation of HSP70 using HSP70-specific siRNA reduced both IAV transcription and replication. The performed immunoprecipitation showed that HSP70 is co-precipitated with the PB2/PB1 heterodimer, thanks to the incorporation of the PA subunit into the PB1/PA or PB2/PB1/PA heterodimer [87].

#### 3.1.4. Severe Acute Respiratory Syndrome Coronavirus 2 (SARS-CoV-2)

The outbreak of the SARS-CoV-2 pandemic in 2019 drew the attention of scientists from around the world to focus on researching and trying to combat it. SARS-CoV-2 belongs to the *Coronavirinae* subfamily of the *Coronaviridae* family. It is a single-stranded, positive RNA virus. Recent sequencing has shown it to be around 29.9 kb in length [117]. SARS-CoV-2 is composed of four structural proteins—S, E, M and N—and sixteen non-structural proteins—NSP1-16 [118]. SARS-CoV-2 causes a disease commonly known as the 2019 coronavirus disease (COVID-19). Many COVID-19 patients develop acute respiratory distress syndrome (ARDS). According to Wu et al. [119] the development of ARDS is responsible for the prognosis in patients, which is exacerbated by the excessive permeability of the endothelium in the lung tissues. Recent studies, however, report that HSP90 inhibitors inhibit endothelial damage and restore endothelial barrier functions [120]. Studies in mice proved that HSP90 inhibitors such as 17-AAG and Luminespib induced a response to unfolded proteins, thereby protecting endothelial cells of the pulmonary aorta and microvessels of the lungs [121].

The S glycoprotein plays an important role during SARS-CoV-2 entry into host cells via the ACE2 receptor, which is located in the host cell membrane. The entry of the virus into the cell takes place by endocytosis or fusion with the surface membrane. The spike of the viral protein binds to ACE2. For the virus to enter the cell, the ACE2-binding spine must be split (activated) by a serine protease similar to 3C-3CLpro for the spike fusion domains to function. The fusion ends with the transfer of the genomic and protein content of the SARS-CoV-2 virus to the cytosol; thus, starting the replication cycle [122].

Alreshidi et al. [123] hypothesized that aspirin-induced HSP70 and HSP90 bind to S-ACE2 and/or AngI, making ACE2 unavailable or at least less accessible to SARS-CoV-2, thereby inhibiting cytokine storms and ARDS. In addition, the research included simulating the docking of selected drugs. Of the more than 10 substances, only piperaquine and aspirin interacted quite efficiently with HSP70/90-AngII compared to HSP70/90-S-ACE2, forming a relatively stable complex. It is worth noting, however, that this experiment was carried out in silico and, therefore, requires validation in in vitro/in vivo tests. Wyler et al. [95] investigated the effect of HSP90 on the replication of SARS-CoV-2 in the Calu-3 cell line, using the inhibitors of HSP90—Onalespib, Ganetespib and 17-(allylamino)-17-demethoxygeldanamycin (17-AAG). Reduced virus yield (about 50–70%) was obtained while maintaining cell viability. RNA sequencing was performed in healthy and infected cells to verify whether the use of HSP90 inhibitors reduced the amount of virus. The results show that in the infected cells, the intracellular RNA of the virus was reduced comparably to the amount of the virus. HSP90 inhibition reduced viral replication and the inflammatory response, particularly of pro-inflammatory cytokines such as IL-6, CXCL10 and CXCL11. The team of Li et al. [124] was also interested in the 17-AAG inhibitor. In their experiment, they proved that 17-AAG strongly inhibits both SARS-CoV and SARS-CoV-2 replication. Another inhibitor of HSP-90 is geldanamycin, the effectiveness of which was confirmed by the team of Sultan et al. [125]. The performed computational analysis of data from RNA sequencing of patients confirmed the inhibition of SARS-CoV-2 growth.

In studies by Joel Selkrig et al. [126] attempts were made to disturb the SARS-CoV-2-induced destabilization of HSP90 by the use of tanespimycin—an HSP90 inhibitor. The resulting inhibition of viral proliferation provided convincing evidence that SARS-CoV-2 requires the presence of HSP90 for proliferation during infection.

### 3.2. DNA Viruses

Table 4 shows collected DNA viruses, which at different stages of infection use the HSP70 and/or HSP90 host. The following subsection focuses on viruses in which both HSP70 and HSP90 are involved.

#### 3.2.1. Human Hepatitis B Virus (HBV)

Human hepatitis B virus (HBV) belongs to the *Hepadnaviridae* family and can cause acute and chronic liver disease. The core protein (HBc) plays an essential role in the viral life cycle, which is organized into two domains: the C-terminal domain that regulates viral replication and the N-terminal domain that is involved in the core assembly [127,147].

In domain C, there is the Cp149 protein which is known to be able to spontaneously form a capsid under appropriate in vitro and in vivo conditions [148]. The capsid is an important structure that protects the viral DNA from external influences. There are few reports of the function of heat shock proteins in capsid formation. Shim et al.’s research [147] proves that for HSP90 to facilitate viral capsid assembly, it must first be activated by p23 and ATP, with which it will form a complex that facilitates the maturation of the client protein. Activated HSP90 facilitates the assembly of the HBV capsid during encapsidation.

In their research, Wook Seo et al. [127] proved that HSP70 synergistically increases the formation of HBV capsid with HSP90. They also theorized that the increased folding resulted from catalyzing the formation of the hexamer core protein. Nevertheless, there is still no confirmation of this theory in the literature. The results on the influence of HSP on HBV capsid formation may lead to the development of new therapeutic strategies for the treatment of HBV infection and the development of hepatocellular carcinoma (HCC) in the future.

#### 3.2.2. Herpes Simplex Virus-1 (HSV-1)

Herpes Simplex Virus-1 (HSV-1) is a DNA-virus which belongs to the *herpesviridae* family. The virus genome is about 152 kb in size, whereas the virion diameter ranges from 150 to 300 nm [149]. It is one of the most common and contagious viruses that produce viral infections in most humans. The virus can be transmitted by contact with a person going through an infection reactivation and shedding the virus, most often when the reactivation in a person is asymptomatic.

HSP90 is crucial for gene expression on many levels such as transcription and translation, among others. It regulates the location of the virus DNA polymerases through relocating them from the cell nucleus into the cytoplasm, where the polymerase is later degraded in the ubiquitin-proteasome pathway when the HSP90 function was inhibited [138].

HSP90 is involved in HSV-1 infection. It plays an important role in the early stages, especially when entering the nucleus as well as during the replication. HSV-1 uses the HSP90 chaperone during infection whereas the viral polymerase may be a client of HSP90. HSP90β participates in the formation of the BALF5 and BMRF1 catalytic subunits complex of the viral polymerase during its transport. HSP90 is also able to inhibit the intracellular nuclear transport of the HSV-1 capsid protein by co-localizing with acetylated tubulin and the capsid protein VP5, where HSP90 inhibitors disrupt its binding to the acetylated tubulin [130].

The HSV-1 virus may be reduced in amount by the AT-522 inhibitor at the exit of the viral nucleocapsid from the nucleus as well as during the expression and translocation of nuclear proteins such as pUL31 and pUL34 [150].

In an HSV-1-infected cell nucleus near the viral replication compartments, virus-induced chaperone-enriched (VICE) foci are formed, which are the locus of a higher concentration of HSP70/HSP40. The HSP90 chaperone system engages HSP70/HSP40 in eukaryotes. HSP90 is found within viral replication compartments as well as in the adjacent to VICE foci. HSP70 and Hsc70/HSP40 are redistributed to those sites. These are also located by the HSV-1 viral portal protein which is a structural component of the viral capsids. The virus possibly creates a mechanism to sequester modified proteins in order to prevent triggering an antiviral response [138].

HSP90 promotes the translation of the conserved herpesvirus protein kinase (CHPK) of HSV-1 as well as of other viruses. CHPK plays a major role in gene expression, virus DNA replication, exiting the capsid nucleus and repair of the damaged DNA [151]. In an infected cell, the HSP subpopulation is in a state of increased activation, similar to what occurs in neoplasm cells. Such a state suggests that HSP90 activation can be a common cell reaction to various stress factors.

HSP70 translocation from the cytosol to the nucleus is activated by the immediate-early viral protein, ICP0, a regulator of viral gene expression. Hsc70, components of the 26S proteasome and virus UL6 portal protein are co-localized, altogether creating a channel for DNA entry and exit from the capsid. UL6 is highly ubiquitinated in the nucleus, indicating that Hsc70 may be responsible for the correct folding and degradation of UL6 in the ubiquitin–proteasome pathway [131].

#### 3.2.3. The Hepatitis C Virus (HCV)

HCV causes hepatitis C, the frequency of which is difficult to estimate due to the long latency period [152]. Structurally, HCV is a small, enveloped virus of the Flaviviridae family. Its positive RNA encodes a polyprotein that is cut by viral and cellular proteases to produce structural and nonstructural proteins [153].

Research by Khachatoorian et al. confirmed the role of HSP70 in HCV replication. They explained this through a knockdown of HSP70 which led to a reduction in virus production. HBD-mediated action of the NS5A/HSP70 complex is involved in the NS5A-dependent translation of the HCV genome via IRES [154]. Gonzalez et al. [155] as well as Lim et al. [156] showed that HSP70 knockdown reduces IRES-mediated translation and protein production. The NS5A/HSP70 interaction is critical for viral proliferation. A study has also been carried out which shows that the related heat shock protein Hsc70 also binds directly to NS5A; however, HSC70 has a different function than HSP70, and is involved in the formation of an infectious virion [157].

As with many other viruses, HSP90 enhances HCV replication by contributing to the regulation of viral polymerase activity. HSP90 stabilizes both the phosphoinositide kinase 1 (PDK1)-dependent kinase and the kinase preceding the NS5 PRK2 phosphorylation kinase, which indirectly affects the activity of NS5 polymerase [158]. HSP90 also influences the maturation of HCV protein kinase 2/3 (NS2/3). After translation, this kinase is cleaved into two separate proteins, which is crucial for the maturation of the NS2/3 protein, and HSP90 is responsible for the correct folding of the newly formed NS2/3 protein [96]. In addition, HSP90, with the help of the FKBP8 gene (folding and transport gene), forms a complex with NS5 and regulates its activity. The inhibition of the HSP90–NS5A complex formation leads to the inhibition of viral replication [159].

## 4. Conclusions

Members of the HSP70 and HSP90 families are involved in viral infections in many different ways. First, they support viruses when entering host cells by forming complexes on the cell surface. They also support viruses during their replication by directly interacting with the viral polymerase. HSP70 and HSP90 are important for viral gene expression. They are involved in assembling the capsid of some viruses. Viruses use the HSP70 and HSP90 proteins to fold their proteins and increase their chances of survival under unfavorable host conditions. A comprehensive knowledge of the use of chaperones during viral infection would provide new insight into viral replication mechanisms and potential therapies. The use of newer and more effective inhibitors directed against HSP70 and HSP90 proves that they are a very good therapeutic target in viral infections.

## Figures and Tables

**Table 1:** HSP70 isoform functions.

Isoform	Location	Function	References
HSPA1	cytoplasm nucleus lysosomes	inhibiting the accumulation of protein aggregates; protection of the mitotic cell against division abnormalities; stabilization of the lysosomal membrane; inhibition of the release of lysosomal hydrolases into the cytosol	[16,17,18,19,20,21]
HSPA1L	cytoplasm nucleus	unknown	
HSPA2	nucleus	chaperone for the cyclin B/cdc2 complex during meiotic cell division orphases for the transition protein 1 and -2 (DNA packaging proteins)	[22,23,24]
HSPA5	endoplasmatic reticulum	facilitates the transport of the newly synthesized protein into the lumen of the endoplasmic reticulum and their subsequent folding	[25,26,27]
HSPA6	cytoplasm nucleus	unknown	
HSPA8	cytoplasm nucleus	maintenance of organization, folding of nascent polypeptides, translocation of proteins across membranes, autophagy mediated by chaperones, prevention of protein aggregation under stress conditions and disassembly of clathrin-coated vesicles	[28,29]
HSPA9	mitochondrium cytoplasm endoplasmatic reticulum	interacting with incoming proteins and helping them to fold properly after transmembrane transport	[30,31]

**Table 2:** HSP90 isoform functions.

Isoform	Location	Function	References
HSP90	cytoplasm, cell nucleus	folding and preventing protein aggregation; stabilization of citrate synthase, rhodanase and protein kinase CK-II; aryl hydrocarbon receptor maturation; abrogates v-Src kinase activity; protection of the kinase against the action of phosphatases; cross-linking of actin filaments; protection of tubulin against thermal denaturation; protection of myosin from heat stress	[53,54,55,56,57,58,59,60,61,62,63]
GRP94	endoplasmatic reticulum	promoting the folding of secretory and membrane proteins; shifting toll-like receptors and integrins; calcium binding	[64,65,66,67]
TRAP1	mitochondrium	maintenance of mitochondrial integrity and protection against mitochondrial apoptosis; protection against cell death caused by overproduction of ROS; preventing protein aggregation in mitochondria and supporting protein folding	[68,69,70,71]

**Table 3:** Utilization of HSP70 and HSP90 of host cells by RNA viruses at particular stages of infection.

Stage of Infection	RNA Virus	HSP70	HSP90	References
Internalization of the virus into the host cell	CAV-9	+	+	[72,73,74,75,76,77,78,79,80]
	DENV	+	+	
	EV-71	+	+	
	JEV	+	nd	
	ZIKV	+	nd	
Virus replication	RSV	+	+	[81,82,83,84,85,86,87,88,89,90,91,92,93,94,95]
	HCV	+	+	
	IAV	+	+	
	MuV	+	nd	
	CDV	+	nd	
	EBOV	+	nd	
	SARS-CoV-2	+	nd	
	VSV	nd	+	
	HPIV-2	nd	+	
	HPIV-3	nd	+	
	SV40	nd	+	
	CHIKV	nd	+	
	EV-71	nd	+	
Protein maturation, formation and virions release	HCV	nd	+	[96,97,98]
	Polio	nd	+	
	Rhinovirus	nd	+	
	Coxsackie	nd	+	
	IAV	nd	+	
Virus gene expression	ZIKV	+	nd	[77,99,100]
	EV-71	+	nd	
	CVB3	+	nd	
Virus assembly	Polio	+	nd	[101,102,103]
	CVB1	+	nd	
	IAV	+	nd	
	Reovirus	+	nd	

Legend: CAV-9—Coxsackievirus A9; CDV—Canine distemper virus; CHIKV—Chikungunya virus; CVB1—Coxsackievirus B1; CVB3—Coxsackievirus B3; DENV—Dengue virus; EBOV—Ebola virus; EV-71—Enterovirus 71; HCV—Hepatitis C virus; HPIV—human parainfluenza viruses; IAV—Influenza A virus; JEV—Japanese Encephalitis Virus; MuV—Mumps virus; RSV—Respiratory syncytial virus; SARS-CoV-2—severe acute respiratory syndrome coronavirus 2; SV40—Simian virus 40; VSV—Vesicular stomatitis virus; ZIKV—Zika Virus;.+—documented use of the protein by the virus; nd—no data.

**Table 4:** Utilization of HSP70 and HSP90 of host cells by DNA viruses at particular stages of infection.

Stage of Infection	DNA Virus	HSP70	HSP90	References
Internalization of the virus into the host cell	ADV	+	nd	[127,128,129,130,131,132,133]
	HBV	+	+	
	HSV	+	+	
	Polyomavirus	+	nd	
	SV40	+	nd	
Virus replication	EBV	nd	+	[134,135,136,137,138]
	HBV	nd	+	
	HSV	nd	+	
Virus gene expression	EBV	nd	+	[73,96,139,140,141,142,143,144,145,146]
	HBV	+	nd	
	HCMV	+	+	
	JCV	+	nd	
	KSHV	nd	+	
	Polyomavirus	+	nd	
	VZV	nd	+	

Legend: ADV—adenovirus; EBV—Epstein–Barr virus; HBV—Hepatitis B virus; HCMV—human cytomegalovirus; HSV—Herpes simplex virus; JCV—John Cunningham virus; KSHV—Kaposi’s sarcoma-associated herpesvirus; SV40—Simian virus 40; VZV—Varicella zoster virus; +—documented use of the protein by the virus; nd—no data.
